# Pedunculated Osteochondroma of Inferior Pubic Ramus: A Report of a Rare Case

**DOI:** 10.7759/cureus.82546

**Published:** 2025-04-18

**Authors:** Devendra Sharma, Mayank Mahendra, Aditya Seth

**Affiliations:** 1 Orthopaedic Surgery, King George's Medical University, Lucknow, IND; 2 Orthopaedics, King George's Medical University, Lucknow, IND; 3 Orthopaedics, Post Graduate Institute of Medical Sciences (PGIMS), Rohtak, IND

**Keywords:** femoribus internus perineal approach, hereditary multiple exostosis, inferior pubic ramus, osteochondroma, pedunculated tumor

## Abstract

Osteochondromas are the most common benign bone tumours, which usually manifest as bony projections covered with a cartilaginous cap and originate from the metaphysis of long bones. However, they can create discomfort and difficulties, particularly in non-traditional places, although they are frequently asymptomatic. An osteochondroma arising from the right inferior pubic ramus is a rare case. A 31-year-old gentleman presented with an enlarged swelling in the right groin for 17 years, giving him great discomfort, difficulty in performing sexual activities, irritation, and aesthetic issues. The swelling was insidious in onset, firm and non-pliable, measuring 15 x 11 cm, and gradually progressive over the years. It displaced the scrotum and was fixed to the underlying bone. This pedunculated bone growth arising from the right inferior pubic ramus was identified by plain X-ray, pelvic anteroposterior and lateral view, and computed tomography imaging. An excisional biopsy was performed on the patient using the femoribus internus perineal approach. The tumour was carefully removed piecemeal using an osteotome. After surgery, radiographs confirmed complete removal. This case study demonstrates the effective application of the femoribus internus perineal approach and excision technique. There was a resolution of symptoms, and no symptom recurrence was noted after surgery. This case illustrates the importance of tailored surgical methods for managing osteochondromas in non-traditional locations.

## Introduction

Osteochondromas represent the most common types of benign bone tumors, comprising 9% of all bone tumors and 20-50% of benign bone tumors [1]. Due to their often asymptomatic nature, the precise incidence of osteochondromas is challenging to determine. These tumors are thought to result from hamartomatous proliferations of bone and cartilage, likely originating from growth-plate cartilage that becomes trapped and protrudes through the cortex. Typically, osteochondromas develop in the metaphyseal region near the physis and are most commonly diagnosed within the first two decades of life. They can present as either sessile or pedunculated growths. Osteochondromas are more frequently found in the lower extremities, particularly on the distal femur and proximal tibia, but they can also occur in other locations such as the proximal humerus, distal radius, and less commonly, at the skull base, vertebral column, ribs, scapula, and pelvis [2]. When located in the pelvis, shoulders, and hips, they have a higher risk of undergoing malignant transformation [3]. Typically, osteochondromas affect bones formed through endochondral ossification, and it is rare for them to originate from bones formed by intramembranous ossification, such as the scapula, pubic ramus, clavicle, and ribs [4]. Here, we present a rare case of a pedunculated osteochondroma arising from an uncommon location, the inferior pubic ramus.

## Case presentation

**Figure 1:**
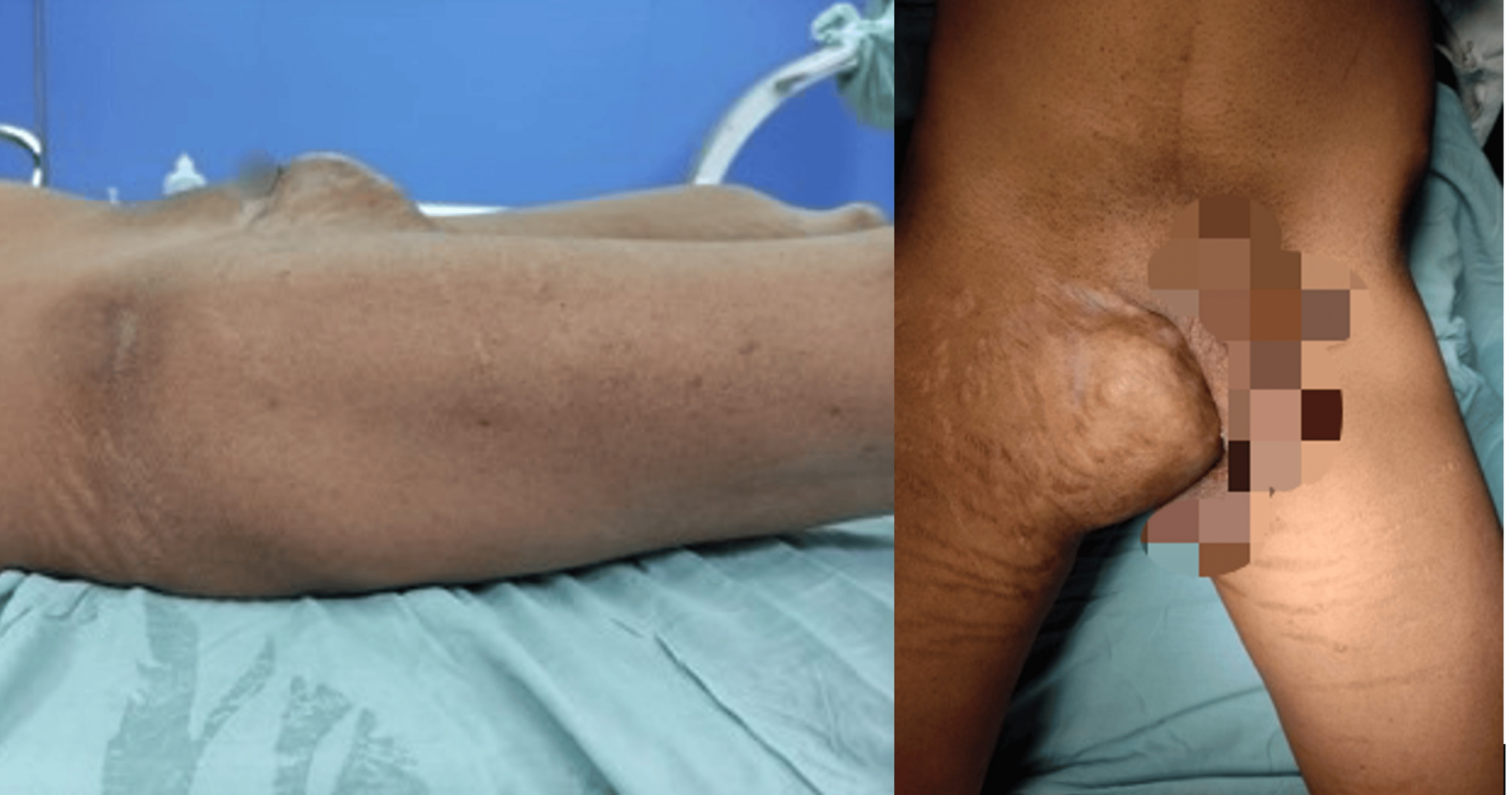
Preoperative anterior (right side) and lateral (left side) view of patient's presentation

A 31-year-old gentleman presented to our outpatient department of Orthopedic Surgery with complaints of swelling in the right groin region for a duration of 17 years. The swelling was insidious in onset; initially, it was small but gradually increased in size over the years. The patient reported experiencing mass effect due to the swelling, including irritation of the overlying skin, difficulty in wearing clothes, and difficulty in performing sexual activities. The patient did not have any urological complaints but has cosmetic concerns (Figure 1). 

On examination, there was a firm to hard swelling approximately 15 × 11 cm in size over the right inguinal region, originating medial to the mid-inguinal point and projecting inferomedially. The swelling was non-tender, non-mobile, and fixed to the underlying bone but not to the skin or overlying tissue. The swelling displaced the scrotum towards the contralateral side, and a cough impulse was absent. The distal neurovascular examination was normal. The patient had multiple swellings at various sites on his body, and the bones of the forearm were bowed, although the patient did not report any significant functional loss.

**Figure 2:**
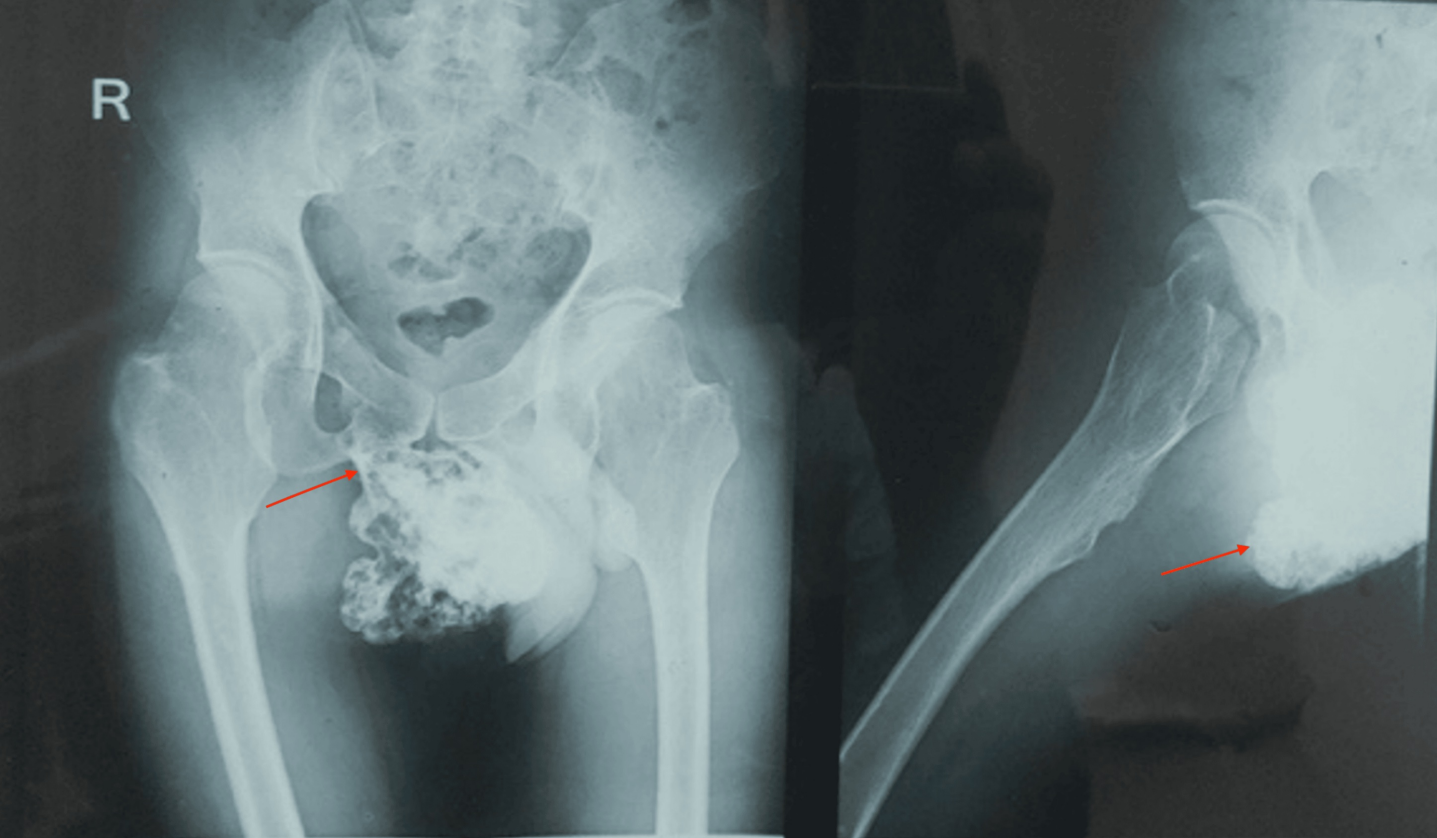
X-ray images of the pelvis Images from anterio-posterior (left) and lateral (right) views showing osteochondroma of the right inferior pubic ramus.

**Figure 3:**
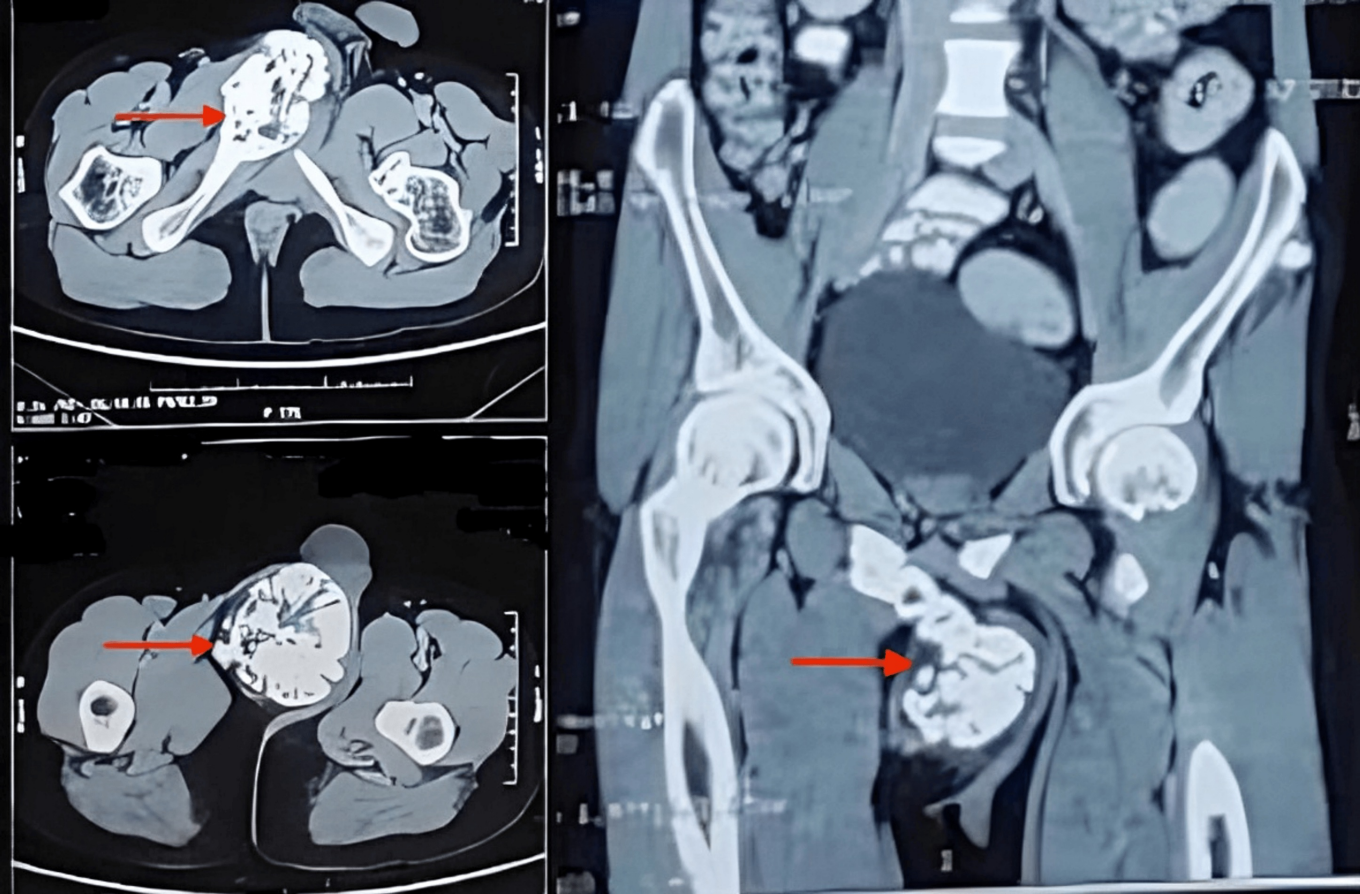
Computed tomography images Axial view (left) and coronal view (right) of the pelvis showing right ischiopubic ramus osteochondroma.

Plain X-rays of the pelvis anteroposterior and lateral view (Figure 2) and CT imaging of the pelvis (Figure 3) revealed a pedunculated bony growth measuring 13 x 9 x 6 cm in transverse, craniocaudal, and anteroposterior dimensions, arising from the right inferior pubic ramus. The growth was roughly round in shape, with its cortex in continuity with the cortex of the right inferior pubic ramus. Due to economic constraints, a magnetic resonance imaging (MRI) could not be performed.

The patient was scheduled for an excisional biopsy via the femoribus internus perineal approach. Under spinal anesthesia and with urinary catheterization, the patient was placed in the lithotomy position. After standard scrubbing, painting, and draping, a 10 cm incision was made from the base of the lateral aspect of the penis along the lateral border of the scrotum towards the ischial tuberosity. The skin, subcutaneous tissue, and deep fascia were incised layer by layer. The adductor muscle group and obturator externus were separated subperiosteally by sharp or blunt dissection from their pelvic attachments, exposing the lateral border of the inferior pubic ramus.

**Figure 4:**
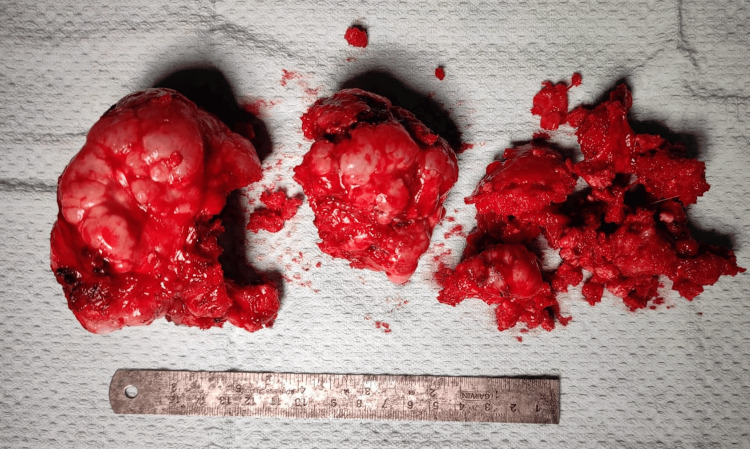
Gross image of the excised mass

The tumor appeared as a whitish-reddish mass with an irregular surface. The nearby neurovascular bundle and urological structures were not involved. The tumor was excised piecemeal (Figure 4) using an osteotome, taking care not to damage the surrounding soft tissue. After removal of the main mass, the stalk was excised from the inferior pubic ramus. The histopathological examination of the excised mass shows mature hyaline cartilage with overlying fibrous perichondrium, enchondral calcification at the bone and cartilage junction, with marrow elements within the stalk. The wound was irrigated with normal saline and closed in layers. The surgery took approximately 55 minutes, with an estimated blood loss of around 50 mL.

**Figure 5:**
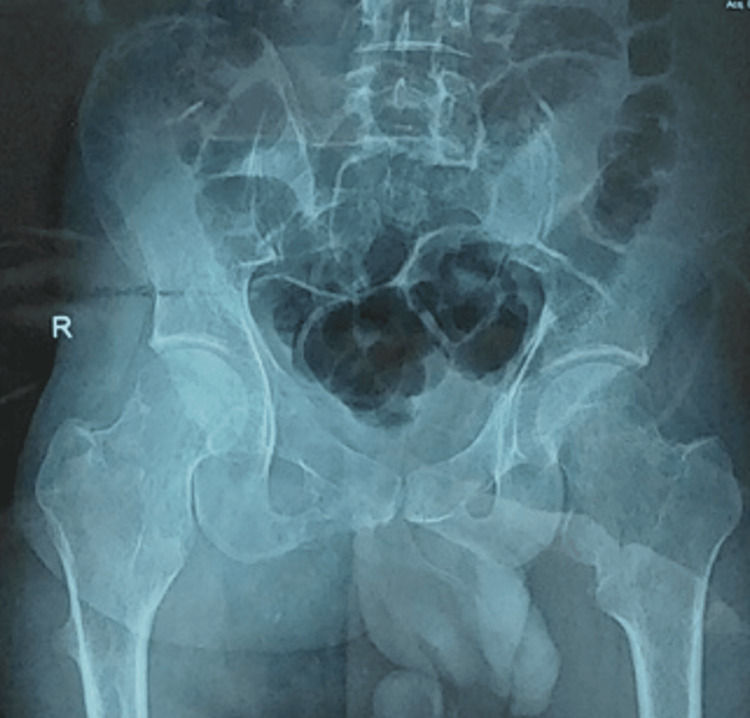
Post-operative X-ray of the pelvis (anteroposterior view)

Postoperative radiographs confirmed the complete removal of the tumor (Figure 5).

**Figure 6:**
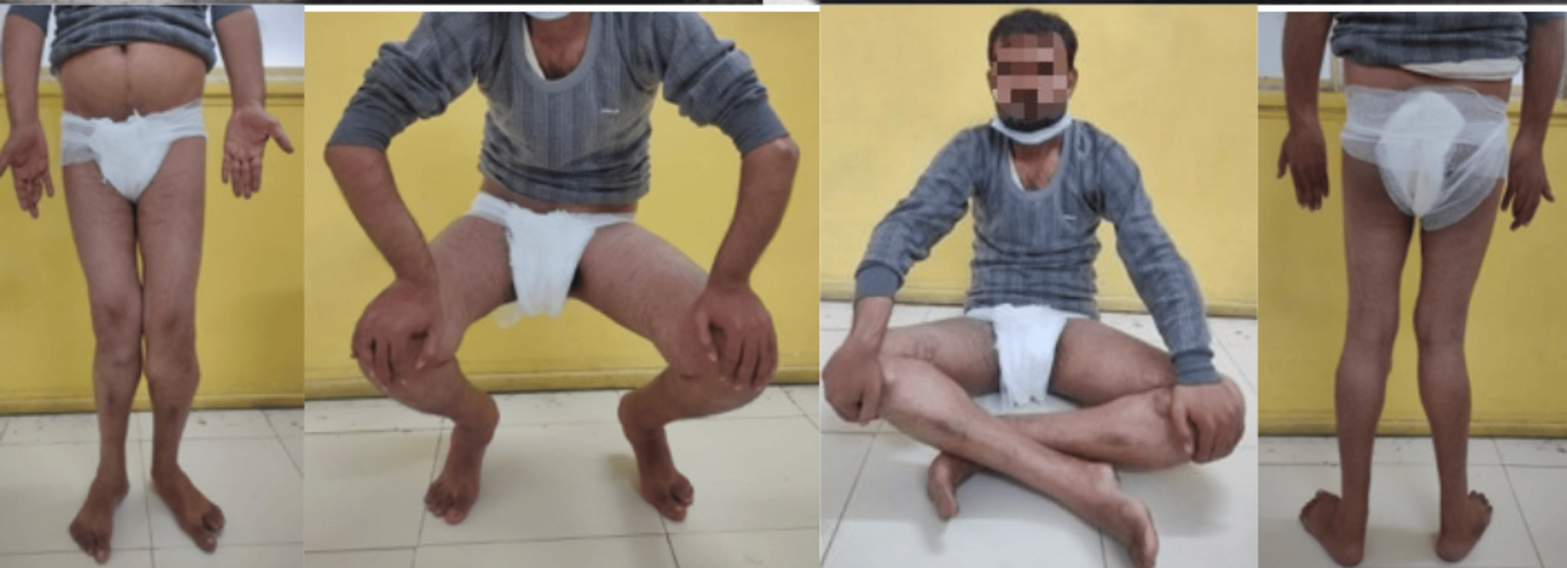
Post-operative image of the patient performing various day-to-day activities

At the three-month follow-up, there was no evidence of recurrence. The patient reported resolution of his complaints, improved psychological outlook due to the enhanced cosmetic appearance, and no difficulties in sexual activities (Figure 6).

## Discussion

Osteochondroma, also known as an exostosis, is a benign osteocartilaginous tumor and is the most common benign bone tumor. Most lesions are identified during the growing years, usually in the first two decades of life, and are more common in males. They are thought to arise from trapped growth-plate cartilage that grows through the cortex via endochondral ossification beneath the periosteum. About 85% are solitary, with the remainder occurring as part of multiple hereditary exostosis syndrome. Multiple osteochondromas become apparent during childhood, whereas solitary osteochondromas are usually diagnosed in late adolescence and early adulthood [5].

Osteochondromas develop only in bones of endochondral origin and arise from the metaphysis near the growth plate of long tubular bones, especially near the knee. Occasionally, they develop from bones of the pelvis, scapula, and ribs, where they are frequently sessile. Osteochondromas arising from the inferior pubic ramus are rare. Clinically, most lesions are solitary and asymptomatic. They present as slow-growing masses that can cause pain if they compress a nearby nerve, have a fractured stalk, or cause inflammation of a nearby bursa. They can be palpated as firm, immobile masses fixed to the underlying bone. Osteochondromas continue to grow until the patient reaches skeletal maturity [5]. 

This disorder is associated with loss-of-function mutations in the *EXT1 *and *EXT2* genes, which are considered tumor-suppressor genes. The products of these genes help in the biosynthesis of heparan sulfate proteoglycans, which play a role in growth factor signaling in the normal epiphyseal plate. Mutations in *EXT1* or *EXT2* result in systemic heparan sulfate deficiency. Whether sessile or pedunculated, the medullary cavity of the parent bone is continuous with the stalk of the lesion, and the cortex of the underlying bone is continuous with that of the stalk. Histologically, the cartilage cap consists of hyaline cartilage, surrounded by a well-defined perichondrium. The stalk consists of cortical and trabecular bone, with marrow spaces between the bone [6].

Lesions found in multiple hereditary exostoses are similar radiographically and histologically to solitary osteochondromas. The risk of malignant transformation is higher (∼5% to 10%) in patients with this condition than in patients with solitary lesions (less than 1%). Sessile lesions are associated with a higher risk of malignant degeneration. Adequate management requires a thorough preoperative workup, including radiological and laboratory studies. A radiograph of the involved site typically shows the lesion's association with the underlying bone. However, CT and MRI are often required to evaluate the overlying cartilage cap, as its thickness in adults is important in predicting malignant transformation. These imaging modalities also better delineate soft tissues than plain radiographs, providing valuable information regarding structures like the neurovascular bundle, which helps in planning surgical resection [7,8].

For our patient, pelvic radiographs and a CT scan confirmed the association of the mass with the underlying inferior pubic ramus. Nonsurgical treatment is preferred for asymptomatic or minimally symptomatic patients who are still growing and consists of nonsteroidal anti-inflammatory drugs (NSAIDs). Surgical excision of an osteochondroma is considered for symptomatic patients not responding to nonoperative treatment, those with significant cosmetic deformity, symptoms secondary to nerve or vascular compression, trauma, or malignant transformation. Surgical management was decided for our patient due to chronic discomfort, increasing problems with sexual activities, and issues related to his attire. Given the rare location of the lesion, resection was performed using a lesser-known femoribus internus perineal approach. The tumor was completely excised, and the patient has been regularly followed up with no recurrence [9].

## Conclusions

Osteochondromas arising from the inferior pubic ramus are rare. Usually presents as a painless and hard mass near joints. Usually asymptomatic, and causes symptoms if swelling may compress nearby structures, like urologic structures, and irritate nerves. Treatment is for symptomatic patients and patients having cosmetic or functional disturbances.
